# Three simple and cost-effective assays for AAC(6′)-Ib-cr enzyme activity

**DOI:** 10.3389/fmicb.2025.1513425

**Published:** 2025-04-25

**Authors:** Shizhou Liang, Wenpin Cai, Ruiben Mao, Mengquan Chen, Xianning Dai, Xiaoli Jin, Wanzhong Kong

**Affiliations:** ^1^Department of Clinical Laboratory, Wenzhou TCM Hospital of Zhejiang Chinese Medical University, Wenzhou, Zhejiang, China; ^2^Department of TCM Science and Research Center, Wenzhou, Zhejiang, China; ^3^Department of Clinical Laboratory, Wenzhou People’s Hospital, Wenzhou Women and Children’s Hospital, Wenzhou, Zhejiang, China

**Keywords:** AAC(6′)-Ib-cr, PMQR, quinolone, antimicrobial resistance, *Enterobacteriaceae*

## Abstract

The enzyme AAC(6′)-Ib-cr belongs to plasmid-mediated quinolone resistance (PMQR), first reported in 2006 and now widely disseminating. Here, we developed three phenotypic methods to detect AAC(6′)-Ib-cr enzyme-producing *Enterobacteriaceae* (APE), two of which are proposed innovatively in this research. These tests are based on the following principles: (i) Matrix-assisted laser desorption/ionization time-of-flight (MALDI-TOF MS) can measure the mass shift of 42 Da resulting from ciprofloxacin acetylation by the AAC(6′)-Ib-cr enzyme. (ii) Co-incubation of ciprofloxacin disks with APE results in inactivation of the drug activity, making it unable to inhibit the growth of the indicator organism. We named this test the quinolone inactivation method (QIM). (iii) Based on the principles of the modified Hodge test, we designed the quinolone Hodge test (QHT). Through exploration of optimal conditions for three methods, we found that MALDI-TOF MS provides the most intuitive results after 1 h of incubation. The interpretability of the QIM and QHT results was significantly improved when the indicator organism *E. coli* ATCC25922 was replaced with a quinolone-slightly-resistant isolate. However, *Proteus mirabilis* was excluded from both QIM and QHT due to its swarming motility. Next, a validation study was conducted using a prospectively collected set of 187 clinical strains, demonstrating 100% specificity (MSM: 141/141; QIM, QHT: 135/135) and 100% sensitivity (MSM: 46/46; QIM, QHT: 33/33) compared to the genotype. In a word, this study presented three simple, efficient, and cost-effective methods for detecting APE, suitable for clinical microbiology laboratories under various conditions for the prevention and control of hospital infections.

## Introduction

Quinolone antibiotics are chemically synthesized drugs with advantages of high blood concentrations, excellent broad-spectrum antibacterial activity, and strong tissue penetration (Ahmed and Daneshtalab, 2012; Ball et al., 1998), making them a preferred agent for empirical treatment of infections (Gupta et al., 2011; Pilatz et al., 2020; Taplitz et al., 2018). They were the second most frequently prescribed antimicrobial drugs after β-lactam (Mercuro et al., 2018; Sanchez et al., 2016; Shapiro et al., 2014), but followed by serious bacterial resistance (Logan et al., 2019; Pitout et al., 2022; Zhao et al., 2024). Researches have shown that bacterial resistance to quinolones is mainly mediated by chromosomal mechanisms, including alterations in drug action sites (DNA gyrase and topoisomerase IV gene mutations) and decreased drug accumulation capacity (loss of membrane porins and overexpression of efflux pumps) (Darby et al., 2023; Pham et al., 2019). Of greater concern were plasmid-mediated quinolone resistance (PMQR) due to their higher transmissibility (Ellabaan et al., 2021), including: Qnr protein families, which protect quinolone targets; the AAC(6′)-Ib-cr enzyme, which acetylates ciprofloxacin and norfloxacin; and efflux pumps mediated by *qepA* and *oqxAB* plasmid genes (Ruiz et al., 2012). Among them, the *aac(6*′*)-Ib-cr* gene is the most threatening as it provides a selective advantage in the presence of ciprofloxacin (Phan et al., 2022). Since its first report in 2006 (Robicsek et al., 2006), the prevalence of strains carrying the *aac(6*′*)-Ib-cr* gene has been increasing in both clinical and environmental settings. According to recent studies, this gene has been detected in various countries of Asia (Al-Khafaji et al., 2024; Kuo et al., 2024; Sohrabi et al., 2024; Zhan et al., 2021), Europe (Hrovat et al., 2019; Mounsey et al., 2021; Piccirilli et al., 2021), Africa (Al-Gallas et al., 2024; Masoud et al., 2021; Swedan et al., 2023), and the Americas (de Oliveira Alves et al., 2022; Golden et al., 2021; Logan et al., 2023) across multiple Gram-negative bacteria. The detection rates of the gene vary significantly by region and source, typically ranging from 10% to 50%. Furthermore, the distribution of the *aac(6*′*)-Ib-cr* gene is widespread, with its presence detected not only in clinical strains but also in animals, food, and water sources (Al-Gallas et al., 2024; Mounsey et al., 2021; Patel et al., 2024; Zahra et al., 2023).

However, unlike the *qnr*, *qepA*, and *oqxAB* screening, which can be accomplished by PCR amplification of the target genes, the detection of *aac(6*′*)-Ib-cr* is more complicated. This is because AAC(6′)-Ib-cr belongs to a variant of aminoglycoside acetyltransferases, and the differences between its encoding gene *aac(6*′*)-Ib-cr* and the wild-type *aac(6*′*)-Ib* exist only on just two nucleotides (Trp102Arg and Asp179Tyr) (Robicsek et al., 2006). These specific point mutations enable the acetylation of norfloxacin and ciprofloxacin at the amino nitrogen on position 7-C of the piperazine ring, thereby reducing drug activity (Vetting et al., 2008; Maurice et al., 2008). Therefore, the screening for *aac(6*′*)-Ib-cr* traditionally involves amplification of the target gene, followed by sequencing or restriction analysis (Pitout et al., 2008). Obviously, this is a time-consuming and expensive work. Although several studies have proposed alternative methods for *aac(6*′*)-Ib-cr* sequencing (Hidalgo-Grass and Strahilevitz, 2010; Wareham et al., 2010), these methods still relied on the use of large equipment not typically available in ordinary laboratories. To date, no study has proposed a method for detecting the AAC(6′)-Ib-cr enzyme based on common antimicrobial susceptibility testing consumables.

Here, we proposed three low-cost phenotypic methods of detecting the AAC(6′)-Ib-cr enzyme in *Enterobacteriaceae*. They each possess distinct characteristics and are suitable for clinical microbiology laboratories under different conditions. We aim to (i) improve existing phenotypic detection methods for AAC(6′)-Ib-cr, (ii) develop and validate QIM and QHT as reliable alternatives, and (iii) compare their performance with mass spectrometry and PCR. These methods are based on the following principles: (i) Matrix-assisted laser desorption/ionization time-of-flight (MALDI-TOF MS) is able to measure the mass transfer of 42 Da resulting from acetylation of ciprofloxacin by the AAC(6′)-Ib-cr enzyme. Based on this, the appearance of spectrum peaks representing acetylated ciprofloxacin and its sodium or potassium adduct reveals the presence of the AAC(6′)-Ib-cr enzyme. This method was initially reported by Pardo et al. (2016), and we followed their steps with some modifications while enriching the species of the measured strains. (ii) The quinolone inactivation method (QIM), which is introduced and named for the first time in this study, is based on a principle derived from the Carbapenem Inactivation Method (CIM) (Pierce et al., 2017; van der Zwaluw et al., 2015): after co-incubation with test strains for some time, the ciprofloxacin adhered onto the disk will be inactivated by the enzyme-producing strains, thereby unable to inhibit the growth of the indicator organism. Furthermore, replacing the indicator organism *E. coli* ATCC 25922 with an isolate with only slight resistance to quinolones can greatly improve the observation of results. Because AAC(6′)-Ib-cr enzyme can easily reduce the quinolone concentration to a level where slightly resistant strains can grow, but it still cannot be lowered enough to support the growth of highly sensitive wild-type strains, as this would require nearly complete depletion of the quinolone. (iii) The last approach is based on the reduction of quinolone drug activity by the AAC(6′)-Ib-cr enzyme-producing *Enterobacteriaceae* (APE), allowing indicator organisms to grow in a curved manner along the inoculum of the tested strain toward the disk. This principle was first proposed by Hodge et al. (1978) to detect penicillinase and has subsequently been modified multiple times for detection of β-lactamases. The excellent performance of Modified Hodge Test (MHT) for screening carbapenase has been repeatedly demonstrated (Lee et al., 2001; Pasteran et al., 2016). In this study, we attempt to apply this principle to the detection of quinolone acetyltransferases, and therefore name it the quinolone Hodge Test (QHT).

## Materials and methods

### Bacterial isolates

(i) Isolates genetically tested: During the first stage of this study, a total of 14 *Enterobacteriaceae* strains were included to explore the optimal conditions for the three methods. All isolates have been previously characterized at the molecular level to determine the various mechanisms of resistance to quinolone antibiotics, including *aac(6*′*)-Ib-cr*, *qnrA*, *qnrB*, *qnrC*, *qnrD*, *qnrS*, *qepA*, as well as the quinolone resistance-determining regions (QRDRs) of the *gyrA* and *parC* genes encoding type II topoisomerases.
(ii) Indicator organisms: We collected four *E. coli* strains for the screening indicator organisms in QIM and QHT, including *E. coli* 28B220 with a single QRDR mutation (*gyrA*: S83L), *E. coli* B2-4 with a single QRDR mutation *(gyrA*: D87N), *E. coli* B2-5 with two QRDR mutations (*gyrA*: S83L; *parC*: S80I), and *E. coli* ATCC 25922. All of these strains were confirmed to not carry any of the aforementioned PMQR genes.
(iii) Prospective clinical isolates: A total of 187 quinolone non-susceptible *Enterobacteriaceae* isolates representing 9 genera, including *E. coli* (*n* = 104), *K. pneumoniae* (*n* = 47), *Klebsiella oxytoca* (*n* = 1), *P. mirabilis* (*n* = 19), *Raoultella ornithinolytica* (*n* = 2), *Serratia marcescens* (*n* = 3), *Enterobacter* spp. (*n* = 3), *Citrobacter* spp. (*n* = 4), *Morganella* spp. (*n* = 3) and *Salmonella* sp. (*n* = 1), were isolated from clinical specimens in Wenzhou Hospital of Traditional Chinese Medicine from March to June 2023. A single isolate per patient was collected. All isolates were re-identified by Matrix-Assisted Laser Desorption/Ionization Time-of-Flight Mass Spectrometry (MALDI-TOF MS; bioMérieux, Lyons, France) and utilized for methodological validation experiments of the three methods in the second stage of the study.

### Antimicrobial susceptibility testing

The MICs of ciprofloxacin and levofloxacin were determined by the broth microdilution method (Nucien Pharmaceutical Co., Ltd. Guangzhou, China) and interpreted according to Clinical and Laboratory Standards Institute (CLSI)^1^ guidelines.

### PCR and sequencing

The test strains and indicator organisms used in this study were all characterized at the molecular level by PCR and sequencing methods to identify quinolone resistance genes, including *aac(6′)-Ib-cr, qnrA, qnrB, qnrC, qnrD, qnrS, qepA*, as well as the quinolone resistance-determining regions of *gyrA* and *parC*. Bacterial DNA was extracted by the boiling method. The amplification reagents and primer designs were obtained from Shanghai Sangon Biotech Co., Ltd (Shanghai, China). Primers for these genes are shown in the Supplementary Table 1 (Kim et al., 2009; Lavilla et al., 2008; Nakano et al., 2019). Positive PCR products were sequenced by Sanger sequencing method in Shanghai Sangon Biotech Co., Ltd. The obtained DNA sequences were analyzed using the Blast program on the NCBI website^2^ to determine the genotypes.

### Exploration of optimal conditions

**FIGURE 1:**
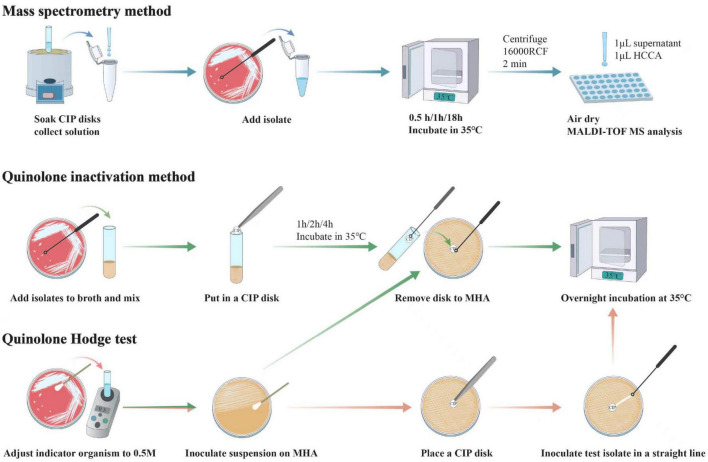
Workflows of mass spectrometry method, quinolone inactivation method and quinolone Hodge test. Each performance of mass spectrometry method and quinolone inactivation method required a concurrent control test which follows the same steps as the regular test, except that the test bacteria are not added. In addition, another variable for quinolone inactivation method and quinolone Hodge test is the four different indicator organisms, which are not annotated in the figure.

In the first stage of the study, we aim to explore the optimal conditions for the three methods and show the experimental steps in Figure 1.

(i) Mass spectrometry method: We followed the method of Pardo et al. (2016) and Oviaño et al. (2017), and made some modifications. First, for reagent preparation, we diluted 100 ml:0.2 g ciprofloxacin injection (Nucien Pharmaceutical Co., Ltd. Guangzhou, China) with normal saline to obtain a concentration of 50 μg/mL. If the number of tests is small, the above solution could be obtained directly by immersing 5–7 ciprofloxacin disks (Kont Biology & Technology Co., Ltd. Wenzhou, China) in 2 mL normal saline. For analysis of bacteria, briefly, using a sterile inoculating loop, one colony was picked and suspended in 100 μL of the aforementioned CIP solution in an Eppendorf tube. Then, the mixture was vortexed for 10 s and incubated at 35°C for 30 min/1 h/18 h (with the incubation time being set as the variable of this method). At the end of incubation, 1 μL of the supernatant obtained after centrifugation was dropped onto a polished steel MALDI target plate, followed by direct overlay with 1 μL of MALDI-TOF matrix [10 mg/mL α-cyano-4-hydroxy-cinnamic acid (HCCA) in 50% acetonitrile and 0.1% trifluoroacetic acid (Bruker Daltonics GmbH, Bremen, Germany)]. The mixture was allowed to dry together. Spectra were acquired using the flexControl 3.3 software on a MALDI-TOF MS instrument (Bruker Daltonics GmbH, Bremen, Germany), with parameter settings: positive ion linear mode, mass range of 300∼600 Da, ion source 1 (IS1) at 10 kV, IS2 at 9.08 kV, lens at 3.00 kV, pulse ion extraction time of 10 ns, and laser frequency at 60 Hz. Additionally, a control experiment was conducted without the addition of any bacteria.

**FIGURE 2:**
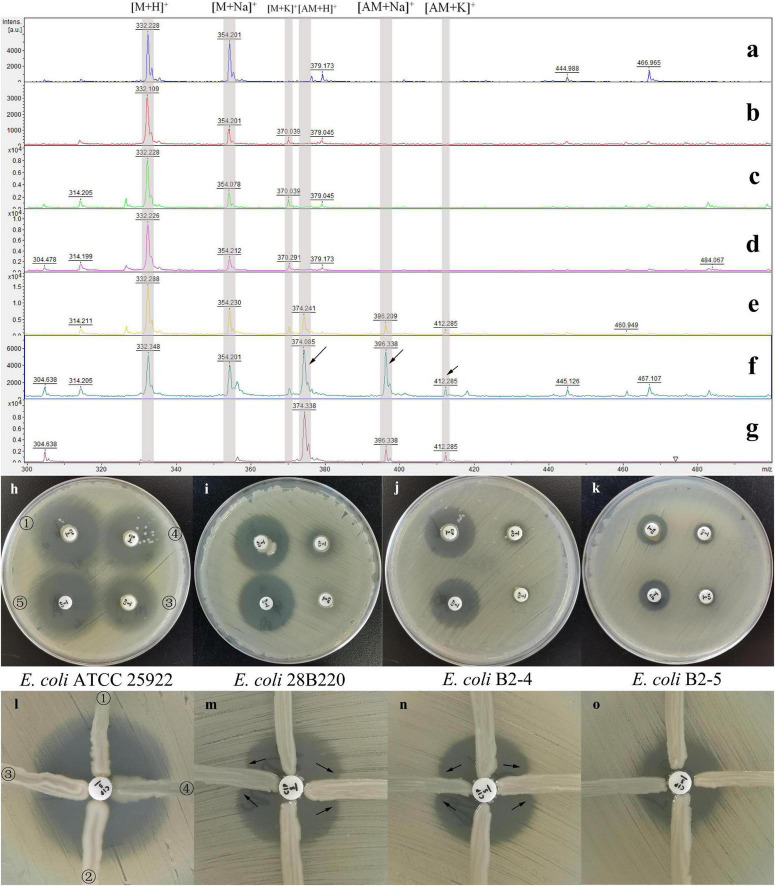
Results of the three methods under different conditions. Results of the mass spectrometry method **(a–g)**, the quinolone inactivation method **(h–k)** and the quinolone Hodge test **(l–o)**. MALDI-TOF MS spectra of ciprofloxacin after 0.5 h **(b)**, 1 h **(c)**, and 18 h **(d)** incubation with NAPE and after 0.5 h **(e)**, 1 h **(f)**, and 18 h **(g)** incubation with APE, and 1 h **(a)** incubation without bacteria. The spectral peaks at 322, 354, and 370 Da represent the three adducts of ciprofloxacin molecular ions: [M+H]^+^, [M+Na]^+^, and [M+K]^+^. The spectral peaks at 374, 396, and 412 Da represent the three adducts of acetylated ciprofloxacin molecular ions: [AM+H]^+^, [AM+Na]^+^, and [AM+K]^+^ (indicated by the arrows). *E. coli* ATCC 25922 **(h, l)**, *E. coli* 28B220 [*gyrA*: S83L; **(i, m)**], *E. coli* B2-4 [*gyrA*: D87N; **(j,n)**] and *E. coli* B2-5 [*gyrA*: S83L, *parC*: S80I; **(k,o)**] were used as indicator organisms. Marked ①, ②, ③, ④, and ⑤ in the figures **(h,l)** are the results of NAPE, NAPE, APE, APE, and control test, respectively, and they correspond to the same positions on the other six plates. In figures **(m,n)**, the curved growth of the indicator organisms on both sides of the test strains, indicated by the arrows, represents that the test strains are enzyme-producing strains.

For data analysis, the relative molecular mass of ciprofloxacin (C_17_H_18_FN_3_O_3_) is 331, and the mass spectrometry peaks for [M+H^+^], [M+Na^+^], and [M+K^+^] appeared at 332, 354, and 370 Da, respectively, as shown in Figure 2. Upon acetylation by AAC(6′)-Ib-cr enzyme, N-acetylciprofloxacin (C_19_H_20_FN_3_O_4_) has gained an additional 42 Da, corresponding to peaks of 374, 396, and 412 Da for these three ionic adducts. Therefore, the presence of these three peaks indicating N-acetylated ciprofloxacin on the acquired spectral graph suggested that the tested bacteria were enzyme-producing strains.

(ii) Quinolone inactivation method: Briefly, using a sterile inoculation loop, 2–3 colonies of test isolate were added to a glass tube containing 1 mL of Mueller-Hinton Broth (Binhe Microbiological Reagents Co., Ltd. Hangzhou, China), followed by vortexing for 5–10 s (control test was performed without adding any bacteria). Next, a susceptibility-testing disk containing 5 μg ciprofloxacin (Kont Biology & Technology Co., Ltd. Wenzhou, China) was gently immersed at the bottom of the suspension and incubated at 35°C for 1 h/2 h/4 h (variable 1). Near the end of incubation, indicator organisms adjusted to 0.5 McFarland turbidity standard were inoculated onto Mueller-Hinton Agar (MHA; Autobio Biological Engineering Co., Ltd. Zhengzhou, China) plates by swabbing. In this study, we tested four indicator organisms (*E. coli* 28B220, B2-4, B2-5, and ATCC 25955, as previously described) as the second variable in the methodology. In addition, the antimicrobial susceptibility phenotypes and genetic characterizations of these organisms are detailed in Table 1. After incubation, the disk was carefully removed from the suspension using an inoculation loop and gently placed onto freshly inoculated MHA plates. The plates were then incubated overnight at 35°C. It is worth noting that when removing the disk, one should avoid dragging it along the inner wall of the glass tube, as condensate water on the wall may dilute the concentration of antibiotics adhering to the paper disks, leading to false-positive results. In addition, the control experiment followed the same steps except that no bacteria were added during incubation, as previously described.

**TABLE 1:** Genotypes, quinolone susceptibility, and QHT results of strains involved in the first stage.

Strain (species)	MIC^a^ (μg/ml)		Resistance mechanism					Mass spectrometry method			Quinolone Hodge test			
			**PMQR**			**QRDR**		**Incubation time**			**Indicator organism (*E. coli*)**			
	**CIP**	**LVF**	***aac(6*′*)-Ib-cr***	***qnr***	***qepA***	***gyrA***	***parC***	**30 mins**	**1 h**	**18 h**	**ATCC 25922**	**28B220**	**B2-4**	**B2-5**
**Test isolate**														
A2 (*E. coli*)	64	32	+	*qnrS*	–	S83L&D87N	S80I	±^c^	+^e^	+	–	+	+	–
A3 (*Enterobacter hormaechei*)	64	16	+	*qnrB*	–	S83I	S80I	+	+	+	–	+	+	–
A141 (*E. coli*)	128	16	+	–	–	S83L&D87N	S80I	+	+	+	±	+	+	–
B24 (*K. pneumoniae*)	64	32	+	*qnrB*	–	S83I	S80I	±	+	+	–	+	+	–
B26 (*K. pneumoniae*)	16	4	+	*qnrB*&*qnrS*	–	wt	wt	+	+	+	–	+	+	–
A63 (*E. coli*)	> 128	32	+	–	–	S83L&D87N	S80I&E84V	±	+	+	–	+	+	–
A67 (*Citrobacter freundii*)	32	8	+	*qnrB*	–	S83T	wt	±	+	+	–	+	+	–
A113 (*P. mirabilis*)	64	16	+	–	–	S83I	S80I	±	+	+	Not applicable assay^d^			
A105 (*E. coli*)	32	32	–	–	–	S83L&D87N	S80I	–	–	–	–	–	–	–
A117 (*K. pneumoniae*)	64	64	–	*qnrB*&*qnrS*	–	S83I&D87G	S80I	–	–	–	–	–	–	–
A102 (*K. pneumoniae*)	32	32	–	–	–	S83L&D87N	S80I	–	–	–	–	–	–	–
6B107 (*E. coli*)	≤ 0.125	≤ 0.25	–	–	–	wt	wt	–	–	–	–	–	–	–
B1-5 (*E. coli*)	0.5	1	–	–	–	S83L	wt	–	–	–	–	–	–	–
A6 (*K. pneumoniae*)	16	32	wt	–	–	S83I	S80I	–	–	–	–	–	–	–
**Indicator organism**														
28B220 (*E. coli*)	0.5 (28)^b^	1	–	–	–	S83L	wt							
B2-4 (*E. coli*)	0.5 (27)	1	–	–	–	D87N	wt							
B2-5 (*E. coli*)	2 (20)	4	–	–	–	S83L	S80I							
ATCC25922 (*E. coli*)	≤ 0.125 (33)	≤ 0.25	–	–	–	wt	wt							

^a^CIP, ciprofloxacin; LVF, levofloxacin; MIC, minimum inhibitory concentration; CIP MICs of ≤ 0.25 is considered susceptible, and ≥ 1 is considered resistant; LVF MICs of ≤ 0.5 is considered susceptible, and ≥ 2 is considered resistant; PMQR, plasmid-mediated quinolone resistance; QRDR, quinolone resistance-determining regions; wt, wild type.

^b^Two values represent the MIC (outside the parentheses) and the Kirby-Bauer method results (inside the parentheses).

^C^±: visually detectable positive results, but not the most readable outcomes.

^d^*P. mirabilis* is excluded from QIM and QHT due to swarming motility.

^e^Shaded cells highlight test results under ideal conditions.

Next day, use a ruler to measure the diameter of the inhibition zone around each CIP disk. If the diameter of the inhibition zone in the test group was smaller than the control experiment by more than 5 mm, it was considered a positive result, i.e., the tested strain was APE. If the inhibition zone diameter was equal to or greater than the control experiment, it was considered as NAPE. If the value was within the range of the above two, it was considered an uncertain result. The selection of this cutoff value was based on the fact that, according to our repeated experimental experiences, conducting the test under optimal conditions resulted in most positive results (inhibition zone diameter) differing from the control experiment by more than 6 mm. Additionally, negative results always exhibited slightly larger diameters than the control experiment.

(iii) Quinolone Hodge test: First, the suspension of the indicator organisms (the four indicator organisms were the only variable for the method) was swabbed on the MHA plate as described previously. Next, a CIP disk was placed on the surface of the plate. Then, using a 10-μl loop, 1–2 monoclonal colonies of the tested bacteria were inoculated onto the plate in a straight line out from the edge of the disk, extending outward for a distance of 20–25 mm. Incubate overnight at 35°C. For the interpretation of the results, the production of AAC(6′)-Ib-cr was considered positive when the shape of inhibition zone on both sides of the tested strain was observed to bend toward the disk. Otherwise, the production of AAC(6′)-Ib-cr was considered negative when the shape of inhibition zone did not change.

### Validation experiment

In the second part of the study, we evaluated the optimized procedure among the 187 blinded clinical isolates previously described. Specifically, under the optimal conditions of the three methods, 187 clinical isolates were tested by MSM, and 168 clinical isolates were tested by QIM and QHT. The prospectively collected strains were directly used for validation tests conducted every three days. Each experiment required one negative control and one positive control, which were selected from the APE and NAPE identified in the first phase of this study. It is worth mentioning that the 19 strains of *Proteus mirabilis* we collected were excluded from both QIM and QHT due to their swarming motility, which caused the entire plate to be covered and prevent observation of indicator results. Subsequently, PCR and sequencing of amplification products were performed to determine the presence of the *aac(6′)-Ib-cr* gene in each strain. Molecular characterization served as the gold standard to calculate the specificity and sensitivity of the three methods, evaluating their performance [Specificity = number of samples with negative results in both the method being evaluated and *aac(6′)-Ib-cr* gene detection / number of samples with negative results in gene detection; Sensitivity = number of samples with positive results in both the method being evaluated and gene detection / number of samples with positive results in gene detection].

## Results

### Antimicrobial susceptibility testing and molecular characterization of quinolone resistance genes

Antimicrobial Susceptibility Testing (AST) and the detection of quinolone resistance genes were conducted on 14 *Enterobacteriaceae* isolates and 3 indicator organisms. Among these, 8 isolates carried the *aac(6′)-Ib-cr gene*, 1 isolate carried wild-type *aac(6′)-Ib*, and the remaining 5 isolates did not carry this gene. Additionally, the 14 isolates were found to carry various combinations of quinolone resistance genes. The molecular characterization of the 3 indicator organisms was also re-validated. The AST results and resistance determinants for the above isolates are detailed in Table 1.

### Mass spectrometry method

MS spectra showed the ion peaks corresponding to the ciprofloxacin molecular ions: [M+H]^+^ at 332 Da, its sodium adduct [M+Na]^+^ at 354 Da, and potassium adduct [M+K]^+^ at 370 Da. The acetylation of ciprofloxacin by AAC(6′)-Ib-cr resulted in a 42 Da shift, with the addition of three acetylation molecule peaks: [AM+H]^+^ at 374Da, [AM+Na]^+^ at 396.35 Da and [AM+K]^+^ at 412 Da (Figure 2).

For the evaluation of experimental variables, the incubation time significantly affects result readability. While a 30-min incubation suffices for visually detectable positive results, we found that a 1-h incubation yields the most readable outcomes, as briefly illustrated in Figures 2e–f. Upon 18 h of incubation, some positive results showed a complete disappearance of spectral peaks representing ciprofloxacin, as depicted in Figure 2g. Additionally, as expected, NAPE consistently lacked spectral peaks of the acetylated form even after 18 h of incubation. Furthermore, in the result of the control test (Figure 2a), the peak at 370 Da representing the ciprofloxacin potassium adduct was absent, whereas it appeared in the experimental groups. We believe that ciprofloxacin was dissolved in NaCl solution lacking potassium ions, which explains the absence of the 370 Da peak. Upon adding bacteria to the solution, potassium ions were partially extruded from bacterial cells into the solution via ion pumps, thereby resulting in weak spectral peaks representing potassium adducts (370 Da and 412 Da).

### Quinolone inactivation method

**FIGURE 3:**
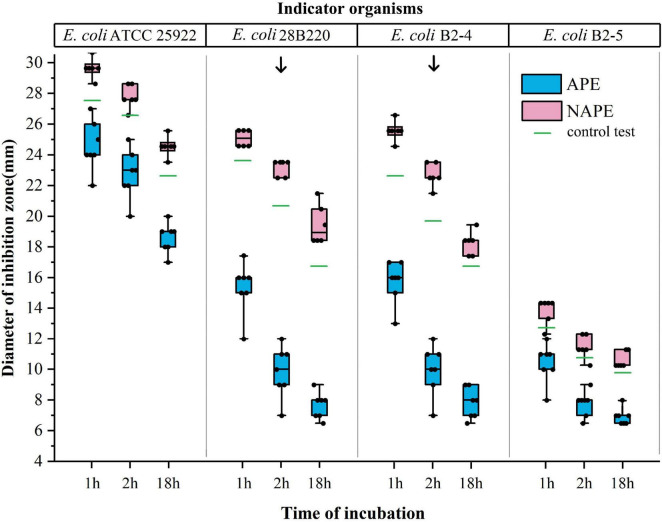
Effect of indicator organisms and incubation time on the inhibition zone diameter in the quinolone inactivation method. The *x*-axis represents the incubation time of the test bacterium with CIP disks, and the *y*-axis represents the inhibition zone diameter of four indicator organisms. Data of the blue boxes are from the results of APE, while data of the red boxes are from the results of NAPE. The green lines represent the results of the control tests. Arrows point to the test results under ideal conditions.

To explore the optimal conditions for QIM, two variables influencing the outcomes were identified: the use of indicator organism *E. coli* 28B220 (*gyrA*: S83L) or *E. coli* B2-4 (*gyrA*: D87N), and the selection of a 2-h incubation time under which all AAC(6′)-Ib-cr enzyme-producing and non-producing isolates were correctly categorized based on the maximum difference in diameter of inhibition zone, as shown in Figure 3.

While studying the feasibility and accuracy of QIM, we observed in preliminary experiments that the indicator organism *E. coli* ATCC25922 was unsuitable for this method. Subsequently, we redesigned three indicator organisms and conducted comparative experiments involving the above four indicator strains in further studies. Through testing 14 isolates, we found that the difference in the inhibition zone diameters of APE and NAPE could not be effectively distinguished when either *E. coli* ATCC 25922 or *E. coli* B2-5 (*gyrA*: S83L, *parC*: S80I) was used as indicator organism, due to the inhibition zones being either too large or too small. However, when *E. coli* 28B220 or *E. coli* B2-4 was used as indicator organism, this difference became significantly pronounced. Moreover, the maximum difference occurred with a 2-h incubation time, as shown in Figures 2h–k.

Considering that the diversity of equipment and consumable specifications may affect the outcomes of this method, we did not set specific inhibition zone diameter values for positive and negative results. Instead, differentiation between positive and negative results was determined through concurrent control testing conducted during each trial. Values were set to ≤ 5 mm compared to the control test for positive results and ≥ the control test for negative results, as previously described. This difference was sufficient to distinguish between positive and negative results. In fact, this difference was above 6 mm for all tests under optimal conditions, as shown in Figure 3. Interestingly, the inhibition zone diameters of all NAPEs were slightly larger than those of the control test. Our explanation for it was that bacterial suspension adhered to the lifted disk, which increased the fluid’s surface tension due to bacterial particulates, resulting in greater absorption of residual antibiotics, a phenomenon that did not occur in the control test (Miller et al., 2017).

### Quinolone Hodge test

We also tested the performance of the four indicator organisms in QHT. The results were similar to those in QIM. When *E. coli* ATCC 25922 was used as the indicator organism, the bending of the inhibition zone was too faint to distinguish between positive and negative results. In contrast, when using *E. coli* 28b220 or *E. coli* B2-4 instead, significantly enhanced growth of the indicator organism was observed on both sides of the APE streak, which was easily recognized by the naked eye. No such phenomenon occurred with NAPE (Figures 2l–o). Additionally, *E. coli* B2-5 was eliminated once again due to its initially small zone of inhibition.

### Validation experiment

The results of PCR and sequencing of 187 clinical isolates used for method performance validation showed that a total of 46 APEs were screened in this prospective study, accounting for 24.6% (46/187), including *E. coli* (12/104), *K. pneumoniae* (18/47), *P. mirabilis* (13/19), *Enterobacter* spp. (1/3), *Citrobacter* spp. (2/4), and no APE was detected in the remaining genera (0/10). All APEs (including strains studied in the first phase) carried the *aac(6*′*)-Ib-cr* gene with both Trp102Arg and Asp179Tyr mutations, which means that no single-point mutation was found across all strains. This finding was consistent with earlier reports (Robicsek et al., 2006). Additionally, 7 isolates carrying the wild-type *aac(6*′*)-Ib* gene were detected. They exhibited identical assay results to non-AAC(6′)-Ib-cr enzyme producers in the validation study. Thus, the proportion of -cr variant among the total number of the gene was calculated as 86.8% (46/53) in this study.

Under optimal conditions, validation experiments were conducted for MSM, QIM, and QHT. The results indicated that MSM correctly identified all APEs and NAPEs with 100% specificity and sensitivity (141/141; 46/46). However, despite QIM and QHT also demonstrating 100% specificity (135/135) and sensitivity (33/33) compared to the genotype, *Proteus mirabilis* was excluded from these data. The data are detailed in Table 2.

**TABLE 2:** Detection of AAC(6′)-Ib-cr enzyme producing clinical isolates using the three methods in this study.

Species (No. of isolates)	MIC range (μg/ml)		No. of positive results		
	**LVF**	**CIP**	**MSM**	**QIM**	**QHT**
**AAC(6′)-Ib-cr-producing Enterobacteriaceae**					
*E. coli* (*n* = 12)	≥ 8	≥ 4	12	12	12
*K. pneumoniae* (*n* = 18)	≥ 8	≥ 4	18	18	18
*P. mirabilis* (*n* = 13)	4– ≥ 8	≥ 4	13	ND	ND
*Enterobacter hormaechei* (*n* = 1)	≥ 8	≥ 4	1	1	1
*Citrobacter braakii* (*n* = 1)	2	≥ 4	1	1	1
*Citrobacter freundii* (*n* = 1)	≥ 8	≥ 4	1	1	1
**non-AAC(6′)-Ib-cr enzyme producer**					
*E. coli* (*n* = 92)	0.5– ≥ 8	0.5– ≥ 4	0	0	0
*K. pneumoniae* (*n* = 29)	1– ≥ 8	0.5– ≥ 4	0	0	0
*P. mirabilis* (*n* = 6)	1– ≥ 8	1– ≥ 4	0	ND	ND
*Salmonella* sp. (*n* = 1)	≥ 8	≥ 4	0	0	0
*Klebsiella oxytoca* (*n* = 1)	≥ 8	≥ 4	0	0	0
*Enterobacter cloacae* (*n* = 1)	≥ 8	≥ 4	0	0	0
*Enterobacter hormaechei* (*n* = 1)	1	1	0	0	0
*Raoultella ornithinolytica* (*n* = 2)	4	2	0	0	0
*Serratia marcescens* (*n* = 3)	0.5–1	0.25–1	0	0	0
*Citrobacter amalonaticus* (*n* = 1)	≥ 8	≥ 4	0	0	0
*Citrobacter freundii* (*n* = 1)	≥ 8	≥ 4	0	0	0
*Morganella morganii* (*n* = 3)	2–4	1–4	0	0	0

ND, not determined (*P. mirabilis* is excluded from QIM and QHT due to swarming motility.); MSM, mass spectrometry method; QIM, quinolone inactivation method; QHT, quinolone Hodge test.

### Performance evaluation and selection guide of methods

We described the specificity, sensitivity, cost, required equipment, turnaround time, and test execution time of four APE detection methods in Table 3. And based on the experience gained from this work, we provide a step-by-step recommendation for selecting genotypic testing, MSM, QIM, or QHT based on existing resources. (i) If MALDI-TOF MS is available, MSM should be prioritized over genotypic testing. This is because the *aac(6*′*)-Ib-cr* gene cannot be detected by a PCR device alone without sequencing equipment. In fact, even in most hospitals of developed countries, only PCR devices are commonly available. More importantly, Phenotypic testing provides a more accurate assessment of clinical resistance compared to genotypic testing, which only detects the presence of *aac(6*′*)-Ib-cr* gene without accounting for the functional expression of it. (ii) In less developed areas, QHT can be chosen as an initial screening method, followed by QIM to recheck samples with subjective controversy in the screening results.

**TABLE 3:** Performance evaluation of different methodologies.

Detection method	Cost per test (equipment)	TAT^a^	Time of perform test^b^	Specificity	Sensitivity	Limitations of method
MSM	< $1.00 (MALDI-TOF MS)	< 2 h	< 0.5 h	100%	100%	MALDI-TOF MS is required in the laboratory.
QIM	< $1.00 (Not required)	18–24 h	< 20 mins	100%	100%	This method is not applicable to *P. mirabilis*.
QHT	< $0.10 (Not required)	18–24 h	< 10 mins	100%	100%	(i) This method is not applicable to *P. mirabilis*. (ii) The interpretation of the results is subjective.
PCR+sequencing	≥ $10.00^c^ (PCR system and sequenator)	1–2 days	> 5 h	–	–	The cost is expensive and the equipment is complex.

^a^TAT: turnaround time.

^b^Incubation time is not included.

^c^The cost of testing varies in different regions.

## Discussion

The resistance phenotype to quinolones is typically insufficient to distinguish between PMQR and other resistance mechanisms (Rodríguez-Martínez et al., 2016; Strahilevitz et al., 2009). As the most prevalent PMQR (Guillard et al., 2014; Jomehzadeh et al., 2022; Shrestha et al., 2023; Tayh et al., 2024), AAC(6′)-Ib-cr confers a low level of quinolone resistance in host organisms, generally below the resistance breakpoints established by CLSI (Machuca et al., 2016; Robicsek et al., 2006; Ruiz, 2019), yet it poses a significantly grave clinical threat (Redgrave et al., 2014; Tacconelli et al., 2018). Several factors substantiate its significance in this process, including its increasing prevalence (Kuo et al., 2024; Zhan et al., 2021), association with other resistance elements (Dulyayangkul et al., 2024; Raherison et al., 2017), as well as the accelerated induction of mutations in QRDRs (Liang et al., 2023; Ortiz-Padilla et al., 2020; Recacha et al., 2019). Of concern is that clinical microbiology laboratories have not yet considered these genes in antimicrobial resistance screening, which may be attributed to the current lack of reliable screening assays for AAC(6′)-Ib-cr (Strahilevitz et al., 2009). In this study, we proposed three methods for detecting the AAC(6′)-Ib-cr enzyme in *Enterobacteriaceae*, all of which demonstrated an overall performance with 100% specificity and sensitivity. Additionally, all three methods cost less than $1 per test, and QIM and QHT did not require complex equipment and professional personnel to perform, as they were easy to operate and yield simple results. Furthermore, our experiments clearly showed that neither mutations in quinolone action target genes nor plasmid-mediated Qnr proteins would interfere with the determination of AAC(6′)-Ib-cr in these new methods. This is not difficult to explain. All tests are based on the acetylation modification activity of the AAC(6′)-Ib-cr enzyme toward ciprofloxacin, which is specific and independent among all mechanisms of resistance to quinolone(Robicsek et al., 2006; Ruiz et al., 2012).

In fact, the concept of using mass spectrometry for the detection of resistant enzymes was already commonplace and dated back to the early 2000s (Burckhardt and Zimmermann, 2011). Currently, it is mainly applied in the detection of carbapenemases (Alvarez-Buylla et al., 2013; Zhang et al., 2023). In contrast to carbapenemases, the detection of the AAC(6′)-Ib-cr enzyme by MS has an additional advantage, because the former result is explained by the complete disappearance of the spectral peaks representing ertapenem (Burckhardt and Zimmermann, 2011), which requires sufficient reaction time, while the latter result is explained by the appearance of new peaks representing the modified products, which are produced early in the reaction (Oviaño et al., 2017; Pardo et al., 2016). Therefore, although the activity of modifying enzymes is much lower than that of hydrolyzing enzymes, the incubation time required for AAC(6′)-Ib-cr detection is still shorter than that needed for carbapenemase detection. In addition, we found that MS was extremely sensitive to CIP detection, as clear ion peaks could be obtained with a 10 μg/ml CIP solution. This was why we believed that CIP reagents could be directly obtained by immersing CIP disks in normal saline. Such adjustments offered numerous benefits: simplifying reagent preparation, reducing costs, and the fact that antibiotic disks are often easy to store for a long time. Furthermore, preliminary research experience showed us that adding the matrix solution directly before the sample droplet dries did not affect the spectral peaks (allowing co-drying). Such a research was necessary because the drying time for sample droplets without surfactants was extremely long at room temperature. Adding the matrix solution could reduce the surface tension of the liquid, similar to the effect of surfactants, and greatly shorten the waiting time for drying (Miller et al., 2017; Wu et al., 2019).

The QIM and QHT methods were proposed for the first time in this study, and our objective was to design a low-cost, as minimally instrument-free method for the detection of the plasmid-mediated quinolone resistance determinant AAC(6′)-Ib-cr in *Enterobacteriaceae*. However, in our previous study (Yunxiang et al., 2015), we found that when *E. coli* ATCC 25922 was used as an indicator organism, it was only suitable for enzymes with strong hydrolytic activity, such as carbapenemases. Its performance was unsatisfactory in detecting *aac(6*′*)-Ib-cr*-mediated acetyltransferase. Therefore, through the comparative experiments involving four strains of *E. coli* with different degrees of QRDR mutations, we attempted to propose new insights into indicator organisms that played a critical role in the test.

The selection of an ideal indicator organism must meet two simple criteria: (i) its minimum inhibitory concentration (MIC) of ciprofloxacin should range between 0.25 to 1 μg/ml. (ii) in the Kirby-Bauer disk diffusion susceptibility test, the indicator organism which must be non-mucoid, should exhibit a clear boundary with an inhibition zone diameter of 28–30 mm. Such organisms, which are readily obtainable from clinical samples, share a common feature of a single-point mutation (S83L or D87N) in the *gyrA* gene, resulting in low-level resistance to quinolones. However, they still exhibit good susceptibility to ciprofloxacin in the Kirby-Bauer test (Machuca et al., 2016; Pham et al., 2019). Our approaches are based on the fact that the enzyme AAC(6′)-Ib-cr produced by the test strains modifies ciprofloxacin, reducing the drug’s effective concentration to such a level that the indicator organism with weak quinolone resistance is sufficient to protect itself, while the highly sensitive wild-type strain (*E. coli* ATCC 25922) remains difficult to survive (Robicsek et al., 2006). This principle is similar to that when a bacterium possesses both a *gyrA* mutation and plasmid-mediated AAC(6′)-Ib-cr enzyme, it always exhibits a higher level of resistance to quinolones (Machuca et al., 2016). We redesigned the indicator organism aiming to combine these two low-level resistance mechanisms to exhibit enhanced drug resistance effects in both QIM and QHT methods. Indeed, The combination of resistance mechanisms is also common to other classes of antibiotics. For example, the resistance of *Pseudomonas aeruginosa* to imipenem is due to a decrease in antibiotic uptake (via downregulation of OMPD2) and the production of AmpC-type β-lactamase (Livermore, 1992). Either mechanism alone is not sufficient to cause clinically significant levels of resistance, but only their combination results in high-level resistance. Therefore, as our experimental results showed, when *E. coli* ATCC25922 was used as the indicator organism, its indication effect for AAC(6′)-Ib-cr production was not significant. However, when it was replaced by strain *E. coli* 28B220, there would be an “illusion” of high level of resistance at the junction of the two bacteria (quinolone Hodge test), similar to that shown when the two resistance mechanisms worked together. When another resistance mechanisms is added, the inherently small inhibition zone may dilute the changes caused by the AAC(6′)-Ib-cr enzyme.

In addition, it was worth noting that the *aac(6*′*)-Ib-cr* genes carried by all 46 strains of APE in this study exhibited identical mutations at codons 102 (Trp-Arg) and 179 (Asp-Tyr). The remaining codons showed high similarity to the wild-type *aac(6*′*)-Ib* gene, and no independent occurrences of the W102R or D179Y mutations were observed. This was consistent with previous studies (Robicsek et al., 2006). We believed that the high sequence homology ensures relatively consistent enzyme activities of the encoded products (Vetting et al., 2008), which was the primary reason for the high performance of these enzyme activity methods. Therefore, compared to labor-intensive, time-consuming and expensive genotype testing, direct detection of AAC(6′)-Ib-cr enzyme activity represents a more cost-effective laboratory strategy. On the other hand, we observed that CIP was completely modified after incubation with APE for up to 18 h (no 322 Da peak was shown; Figure 2g). This compelled us to reassess the AAC(6′)-Ib-cr enzyme. To our knowledge, the concept of AAC(6′)-Ib-cr as a low-level resistance mechanism is based on *in vitro* experiments (Robicsek et al., 2006), which are inherently short-term studies. Thus, its prolonged presence in the human body and its sustained impact on antibiotic efficacy warrant re-evaluation.

There are some limitations to our study. Although all three methods demonstrated 100% specificity and sensitivity, real-world variability should be acknowledged. For example, we are still unable to explain whether a single mutation of the *aac(6*′*)-Ib* gene (W102R or D179Y) in strains might challenge these methods, whether our methods are applicable to non-fermenting Gram-negative bacilli, whether severe porin protein loss that prevents ciprofloxacin from entering the periplasmic space could lead to false negatives, and the variability between different laboratories.

In summary, this study presents three simple, efficient, and cost-effective methods for the detection of AAC(6′)-Ib-cr in *Enterobacteriaceae*. Each method has its own advantages: MSM is a rapid and highly accurate method for AAC(6′)-Ib-cr detection, but it relies on MALDI-TOF MS. QIM and QHT offer cost-effective alternatives but are unsuitable for *P. mirabilis*. Overall, these methods can enhance routine clinical screening for PMQR mechanisms.

## Data Availability

The original contributions presented in this study are included in this article/Supplementary material, further inquiries can be directed to the corresponding authors.

## Footnotes

1
http://www.clsi.org

2
http://blast.ncbi.nlm.nih.gov/blast.cgi
