# Supplementary material for: Three simple and cost-effective assays for AAC(6′)-Ib-cr enzyme activity
Source: Front Microbiol. 2025 Apr 25;16:1513425. doi: 10.3389/fmicb.2025.1513425 (PMC12061968; doi:10.3389/fmicb.2025.1513425)

Quinolone Inactivation Method Operating Procedure Manual

1. Prepare several sterile glass tubes containing 1 ml of Mueller-Hinton Broth.

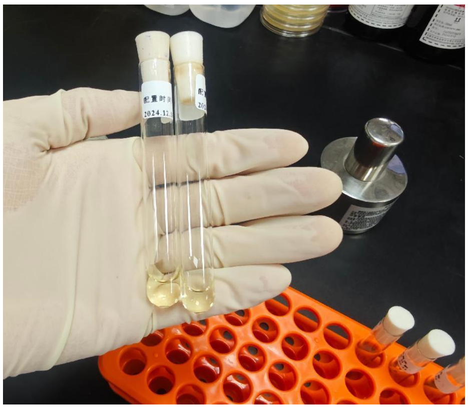

2. Use a sterile inoculating loop to pick 2-3 Colonies of the tested strain and add them to the above solution. Tubes used for control experiments were not supplemented with any bacteria.

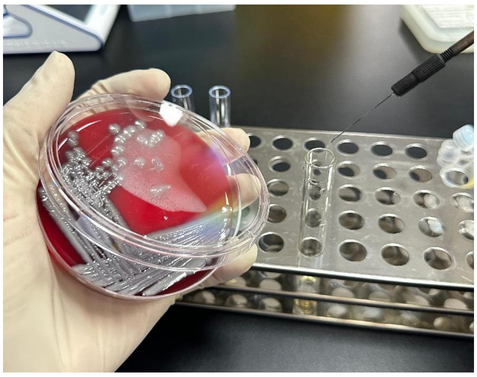

3. Vortex tubes for 5-10s to resuspend the bacteria.

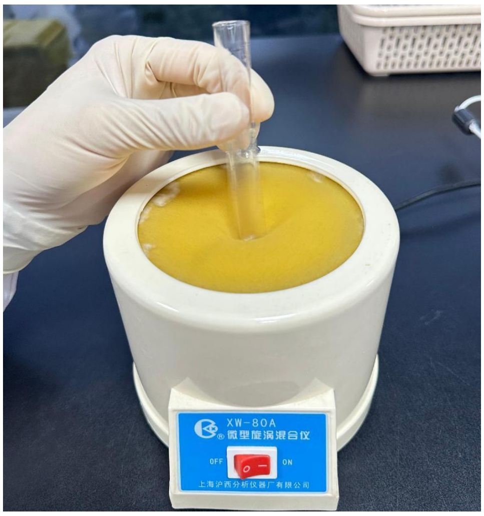

4. Use sterile forceps to place susceptibility testing disks containing 5 μg ciprofloxacin into the tubes, and gently shake the tubes to ensure the disks settle at the bottom. Note: This step should not involve vigorous shaking.

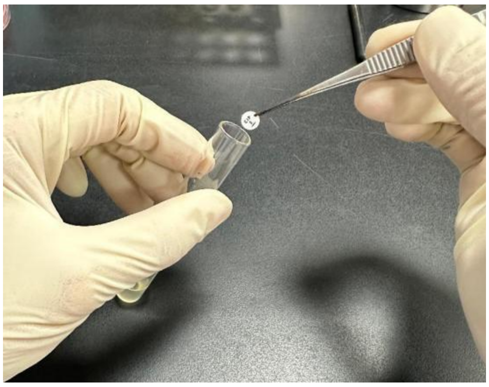

5. Plae the tubes in the incubator at 35°C and incubate for 2 hours.

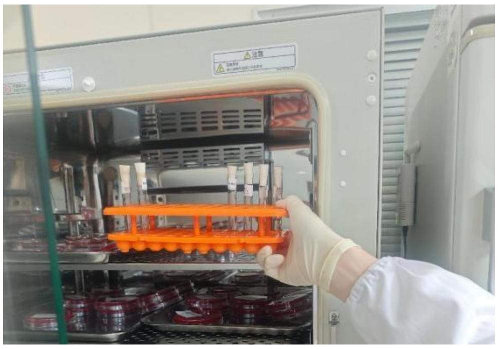

Perform steps 6-7 during the last 10-20 mins before the incubation is complete.

6. Pick a small number of the indicator organism colonies and add them to a 0.45% NaCl solution, adjusted to approximately 0.5 McFarland turbidity standard.

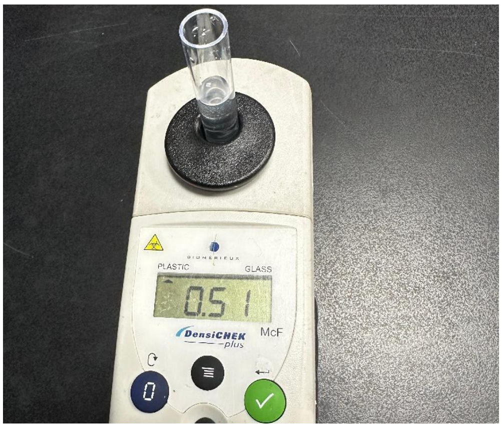

7. Use a sterile cotton swab to dip into the above bacterial suspension and evenly swab it onto Mueller-Hinton agar (MHA) plates.

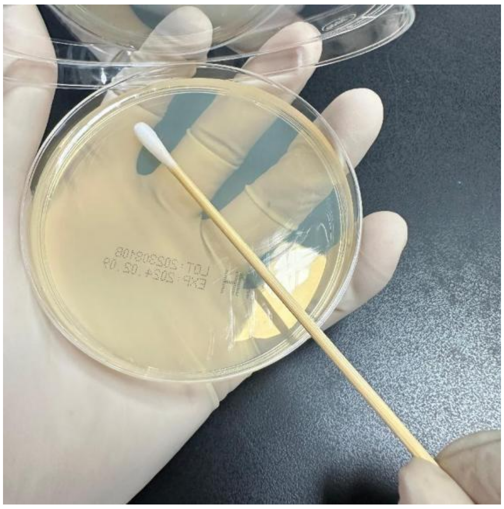

8. After the incubation time is complete, use a sterile inoculating loop to remove the ciprofloxacin disks from the bacterial suspension. Note: Do not drag the disks along the inner walls of the glass tubes, as condensate water on the wall may dilute the concentration of antibiotics adhering to the paper disks.

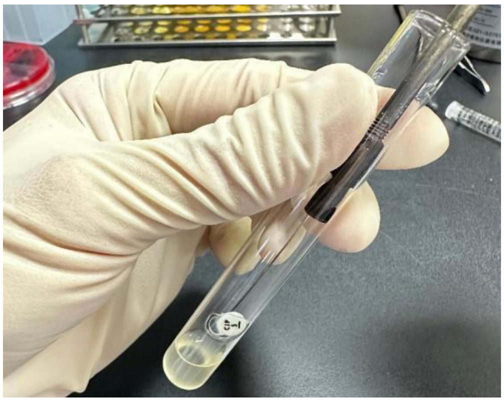

9. Gently place the removed disks onto the above MHA plates.

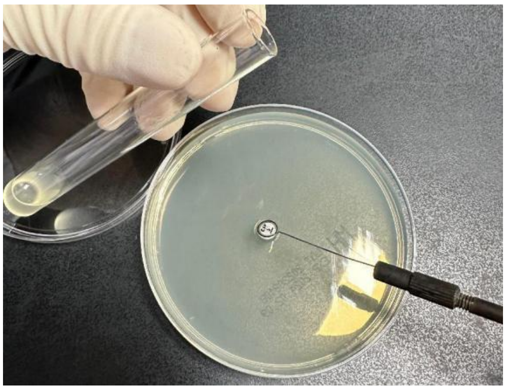

10. Place the MHA plates in the incubator at 35℃ for overnight incubation.

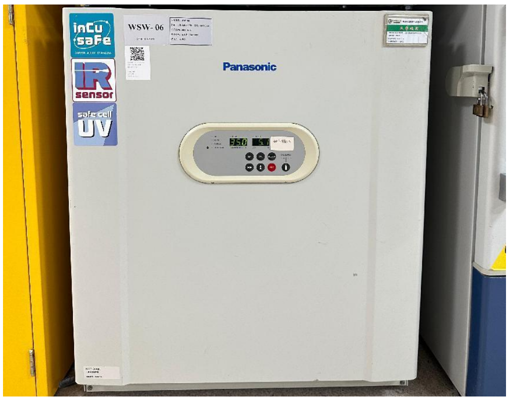

Quinolone Hodge Test Operating Procedure Manual

1. Prepare the MHA plates according to steps 7 and 8 of the Quinolone inactivation method.

2. Use sterile forceps to gently place a ciprofloxacin disk onto the freshly inoculated MHA plates.

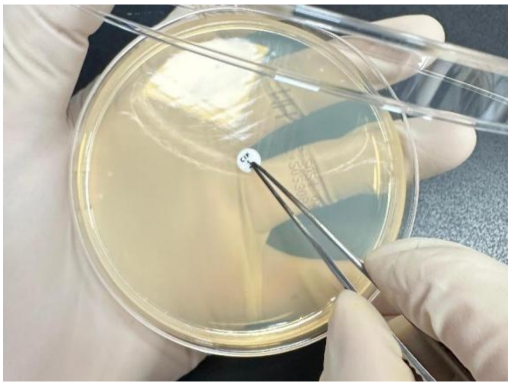

3. Use a sterile inoculating loop to pick 1-2 colonies of the test strain and inoculate them onto the plate in a straight line out from the edge of the disk, extending outward for a distance of 20-25mm.

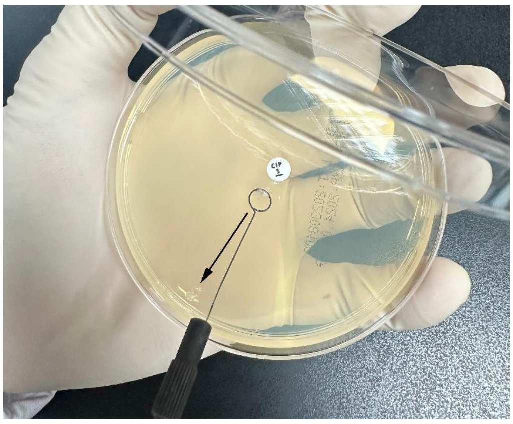

4. Place the inoculated MHA plates in the incubator at 35°C for overnight incubation.

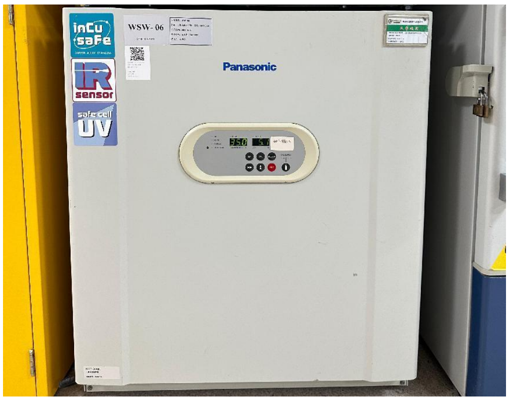

Supplement: Supplementary file 1 [file Data_Sheet_1.pdf]
